# Discussion

**Published:** 1898-08

**Authors:** 


### DISCUSSION.

DR. W. C. DUGAN: I disagree as to early operative interference in fistulæ; we should give them time, cleanse them thoroughly with peroxide of hydrogen, and wait for the ligature to come away, since most of them are due to that cause. If the discharge is copious, thus impairing health, the tract should be opened, the pus cavity curetted, and the cause of the sinus removed. It is not advisable to pack fistulæ tightly with gauze, as this interferes with healing.

If a fistula is high up in the alimentary tract, involving the small intestine, shown by the presence of chyle and absence of fecal odor or feces, the condition demands immediate operation. Here the matter can not be delayed, because the patient will soon die from inanition, and the only operation in such a case is to expose and close the opening in the intestine. The method advised by Senn, sometimes spoken of as Senn's operation, is a good one, since you do not open the cavity until the viscus is cleared, but make a free incision carefully down to the opening into the viscus, and dissect loose its mucous membrane and invert it before any attempt is made to open the peritoneal cavity; then separate the muscular and the submucous coats, putting in a second row of sutures, and you may then open the cavity without subjecting the patient to the risk of infection by feces, as would otherwise be true.

In regard to fistulæ of the large intestine, shown by the fecal odor and passage of feces, the absence of chyle, etc., our treatment should be governed by the condition with which we have to deal. If simply one side of the intestine is attached to the abdominal wall, with only a small quantity of feces being discharged, then you may cut down and dissect the mucous membrane loose, invert it and close as previously mentioned. If most of the feces pass out through the fistula, being practically an artificial anus, it is best to open the peritoneal cavity, because attachments will be found which demand separation. An acute angle of the bowel if not corrected would produce acute intestinal obstruction subsequently. We must do away with the spur in the latter case, and this is best done by thorough separation of the gut. Most fistulæ close if kept clean and let alone. I have met with but few cases, all of which closed within a few weeks except one. This was of the small bowel and the patient ran down rapidly. We resected but the patient died of exhaustion. We waited too long for the operation.

DR. JAMES B. BULLITT: Doctor Morris, who was here not long ago, was the first to speak of a plan of gauze drainage which struck me as being an excellent one, and one which would avoid the difficulty mentioned in Doctor Sherrill's paper in connection with gauze drainage, viz.: that when a cavity is packed with gauze which is left for a long time or even for a few hours, granulations always grow into the meshes of the gauze, and when it is withdrawn it is done sometimes at the expense of certain amount of injury to the surrounding structures. Doctor Morris suggested that he had been in the habit of employing gauze for drainage, at the same time to produce pressure, by taking a wick of gauze and wrapping it with a piece of rubber tissue, this being put down into the cavity and serving for the purpose of drainage and also making more direct channel for fluid to escape, preventing the growth of granulations into the mass of gauze itself. It seems to me this would be advantageous. Perhaps, also, in making gauze for packing into wounds for pressure the suggestion made some time ago might be feasible, that gauze which had been impregnated with rubber tissue should be used. This is probably the best material that can be found for packing for the purpose of preventing hemorrhage by compression, not serving as a drain, but by mechanical pressure serving to control hemorrhage and prevent the after result of the growth of granulations into masses of gauze, rendering it more difficult to be withdrawn in the first place, and in the second place producing after hemorrhage and sometimes after injury.

DR. A. M. VANCE: I have had but little experience with fistulæ, having had only two, one a fecal fistula after an operation for appendicitis, the other a vesical fistula after injury to the bladder. Both healed spontaneously. I agree with Doctor Dugan that time ought to given if it can be done with safety to the patient, as many of these fistulæ will close spontaneously. Radical operation in dealing with an ordinary fistula, the result of an infected ligature, is no small matter. I have never had it to do, but have known some cases where operation was very tedious and exceedingly difficult to perform. I take it that anything beyond curettement under these circumstances would be hazardous. The ligature will usually come away inside of three months. This is the longest time I have known a fistula to remain open from a ligature. I have never had such a case myself, but remember one in the practice of another physician in which I was the consultant, where three months after an abdominal operation the ligature was passed per rectum.

DR. LOUIS FRANK: In my opinion there is but one class of fistulæ which require early operative interference, viz.: the class spoken of by Doctor Dugan, a fecal fistula high up in the intestinal tract, where there is danger of the patient starving to death. I have met with a few fistulæ, also a few sinuses, after surgical operations. The ordinary small fecal fistulæ which follow operations for appendicitis will usually close themselves. I remember to have seen only one fecal fistula which did not close, and that followed an operation for a tuberculous pyosalpinx. This was due to tuberculous infection, the patient being distinctly tuberculous, there being a tuberculous infection along the entire fistulous tract. I recall another case where a fecal fistula occurred after an operation which I performed. Doctor Cartledge afterwards operated upon the fistula, which even then did not close at once but persisted for a long time, though finally it did close. Fistulæ which occur following infected ligatures will close as soon as the ligatures come away. It is merely a question of time, and the danger attending an operation for the cure of the fistula entirely outweighs the good which may be promised.

Nearly all of the cases of sinus, that is, with an infected ligature at the bottom, are due to improper management of drainage rather than to imperfect technique at the time of the operation. Infection often occurs from imperfect management of the drainage tube, or gauze employed in drainage, rather than infection from the ligature. There are cases which appear to be septic where probably the ligature may have become infected at the time used.

I do not believe that sinuses should be treated by packing, neither should fistulæ. I recall one case of fecal fistula which which was treated by packing, and the more it was packed the larger it became. It was finally discovered that a large piece of gauze had gotten into the intestine and had been lying there for quite a while, thus preventing healing of the opening. All of the gauze packing was removed, and the wound closed. It is sometimes advisable to inject stimulating substances, such as balsam Peru, in preference to hydrogen peroxide, which latter will destroy leucocytes and granulations.

DR. A. M. VANCE: I would like for Doctor Sherrill in closing to state whether as a rule a fecal fistula results from a primary wound in the intestine, that is, a wound at the time of the original operation; or is it due to weakening of the gut and subsequent sloughing. An opening left primarily usually causes fatal peritonitis.

DR. J. G. SHERRILL: In one case of fecal fistula which came under my observation I have every reason to believe there was a wound of the intestine at the time of the operation which was not closed. The patient died on the eighth day. In my opinion, most of the cases of fecal fistula occur from an abrasion of the peritoneal surface of the wall of the bowel at the time of the operation, and this abrasion causes the walls of the gut to give away; this is probably often brought about by pulling away gauze which has become attached to this abraded spot. Or perhaps a glass drainage tube may have rested against this abraded spot, although I do not believe a great many fistulæ can be attributed to the effects of a drainage tube. However, an injured intestine may be opened by pressure from a glass drainage tube. Those cases with which I have come in contact have been so persistent and troublesome that I desired to get the opinion of the members of this society upon the subject, and it was with this view in mind that my paper was prepared.

I would like to know exactly when to say operate for a sinus which refuses to heal. Of course I would not ordinarily advise operating upon a sinus under six or eight months, but after it has persisted nine months something ought to be done to relieve the patient of the distressing symptoms which are frequently present. I recently had under my care a patient who came to me suffering with a sinus which refused to heal; this patient was operated upon early last spring, making the length of time that the sinus had existed nine or ten months. Every few weeks this woman has a sense of fullness in the pelvis, great pain and distension, with only a little discharge from the sinus, and evidently a damming-up of the pus, probably from contraction of the fascis in abdominal wall. The discharge distends the fistulous tract so that it will open spontaneously, or it must be opened; then the woman will have comparative comfort for a short time, when the same condition recurs. The case gave me a great deal of trouble and annoyance, and she desired that something be done for her relief. I have postponed operative measures until I feel that the time has about arrived when the sinus should be operated upon. The tissues around the fistulous tract have become markedly thickened and hardened, and, in my opinion, after this length of time has elapsed, even should the ligature come away, the walls of the tract will not coalesce and the sinus will remain patulous.

I am not in favor of much curetting for the relief of simple sinuses, because injury is apt to be done the intestine if a coil happens to lie close to the sinus, and in that case you will have a simple sinus converted into a fecal fistula, which I consider a much more grave condition.

Cases of fecal fistula that are short, where gut lies up against the interior abdominal wall, will in a great many instances get well; but where it runs down deep into the pelvis you will find the fistula to exist a long time and probably not recover until operative measures are instituted, and that the patient will die without an operation. If an operation is undertaken it should not be completed until the opening is found and sutured, no matter what length of time it takes, provided the patient is in condition to withstand prolonged operation.

